# Complementary and Alternative Therapies Targeting Inflammasomes for Human Diseases

**DOI:** 10.1155/2020/7949545

**Published:** 2020-06-09

**Authors:** Young-Su Yi, Tae Jin Lee

**Affiliations:** ^1^Department of Life Sciences, Kyonggi University, Suwon 16227, Republic of Korea; ^2^Department of Neurosurgery, University of Texas Health Science Center at Houston, Houston 77030, USA

Inflammation is an innate immunity protecting the body from invading pathogens and intracellular danger signals, whereas chronic inflammation, that is, slow, repeated, and long-term inflammation has been considered as a major risk factor to induce a variety of human diseases, such as inflammatory, autoimmune, metabolic diseases, and even cancers. An inflammatory response consists of two major consecutive steps: priming and triggering. Priming is the process that prepares for inflammatory response by upregulating the expression of inflammatory molecules, whereas triggering is the process that boosts the inflammatory response by activating the inflammatory molecules. Inflammasomes are intracellular protein complexes consisting of intracellular pattern recognition receptors (NLRP1, NLRP3, NLRC4, NLRP6, AIM2, and caspase-4/5/11), bipartite adaptor (ASC), and other inflammatory molecules, and inflammasome activation is the cardinal feature of triggering during inflammatory responses, which results in caspase-1 activation, gasdermin D-induced pyroptosis, an inflammatory form of cell death, and the secretion of proinflammatory cytokines, IL-1*β*, and IL-18.

Many efforts have been recently made on developing the efficacious and safe anti-inflammatory therapeutics treating various human diseases, and complementary and alternative medicines (CAMs) have been successfully proposed as the effective and safe anti-inflammatory agents that can overcome the serious adverse effects of conventional drugs, such as drug-failure patient groups, side effects, and toxicity. Despite these efforts, a large number of previous studies have mainly focused on the investigation of priming rather than triggering of inflammatory responses. Therefore, the studies focusing on the development of promising CAMs that can treat various human diseases by targeting inflammasomes are recently receiving high attention and required for further investigation. Given this limitation, not only basic studies of CAM-mediated anti-inflammatory actions by targeting inflammasomes but also development of novel CAMs that are selectively targeting inflammasomes, as well as being more efficacious and safer than conventional drugs, is highly demanded.

In this special issue, we invited investigators to contribute the latest original research and review articles investigating *in vitro*, *in vivo*, nonclinical, and clinical/translational studies focusing on the anti-inflammatory effects of CAMs by targeting inflammasomes and potential CAM therapeutics that will help in understanding the basic mechanisms as well as the development of promising CAMs targeting inflammasomes to prevent and treat various human diseases. In this special issue, six original and review articles were published regarding the complementary and alternative therapies targeting inflammasomes for human diseases.

The research article by S. Li et al. investigated whether exercise preconditioning (EP) ameliorates the rat cardiac dysfunction induced by exhaustive exercise by modulating NLRP3 inflammasome pathways and suggested that EP protects the heart from exhaustive exercise-induced injury via downregulation of TXNIP/TRX/NF-*κ*Bp65 and NLRP3 inflammatory signaling pathways. Additionally, this study demonstrated that moderate intensity EP has the best protective effect.

The research article by Y. Geng et al. investigated the underlying anti-inflammatory mechanisms in Delta-opioid receptor (DOR) activation and electroacupuncture-mediated neuroprotection in cerebral ischemia/reperfusion (I/R) injury and suggested that the neuroprotective efficacy of electroacupuncture on cerebral I/R injury may be related to the inhibition of DOR-BDNF/TrkB inflammatory pathway and IL-1*β* secretion, an inflammasome-related proinflammatory cytokine.

The research article by T. Zheng et al. investigated the expression patterns and prognostic characteristics of inflammasome-related genes (IRGs) across cancer types and the development of a robust biomarker for the prognosis of kidney renal clear cell carcinoma (KIRC). This study suggested that the pan-cancer analysis provided a comprehensive landscape of IRGs across cancer types and identified a strong association between IRGs and the prognosis of KIRC. Moreover, this study also suggested that further IRGs signature not only represented a reliable prognostic predictor for KIRC but also verified the prognostic value of inflammasomes in KIRC, contributing to our understanding of therapies targeting inflammasomes for human cancers.

The review article by S. Li et al. classified alkaloids according to structural types and discussed the studies investigating the plant sources, applicable diseases, as well as anti-inflammatory mechanisms by inhibiting the production of IL-1*β*, an inflammasome-activated proinflammatory cytokine of 16 kinds of alkaloids commonly used in clinical treatment, such as berberine, tetrandrine, and stephanine, with the aim of providing a reference for drug research studies and clinical applications. This review provided the insight into the anti-inflammatory mechanisms of alkaloids in Chinese Materia Medicas through suppression of IL-1*β* production, in order to provide a reference for screening active ingredients with anti-inflammatory effects and finding new therapeutic targets.

Another review article by Y. Liu et al. discussed the studies investigating the potential benefits of drugs targeting the NLRP3 inflammasome in the therapy of atherosclerosis and concluded that using drugs targeting NLRP3 inflammasome for treating atherosclerosis is promising, but it also needs further pharmacological studies to verify the efficacy as well as further experimental epidemiological studies to ensure safety.

Finally, the review article by X. Yan et al. discussed the current knowledge on the involvement of cathepsins in the regulation of the innate and adaptive immune responses associated with the inflammatory molecules, IL-1*β* and caspase-1, during inflammasome activation in periodontitis. This review provided the insight given the roles of cathepsins in inflammatory responses that the regulation of cathepsins will be helpful for managing inflammatory responses via modulating the functions of IL-1*β* and caspase-1, inflammatory molecules activated in the inflammasome signaling pathway in patients with periodontitis, and thus beneficial to prevent and relax the systemic diseases as well as neurodegenerative diseases in the global aging society.

We hope that readers will be interested in understanding the mechanisms of CAM-mediated anti-inflammatory effect by inhibiting inflammasomes in inflammatory responses and developing novel promising anti-inflammatory CAM therapeutics that can target inflammasomes to prevent and treat various human diseases. We also hope this special issue attracts the interest of the scientific community, thereby contributing and improving further investigations leading to the discovery of new strategies, the unknown inflammasome targets, and the novel CAM therapeutics for the prevention and treatment of various human diseases.

